# Socioeconomic status and outcomes in critical illness: an unequal burden

**DOI:** 10.62675/2965-2774.20260130

**Published:** 2026-09-23

**Authors:** Sristi Patodia, Larissa Bianchini, Carla Marchini Dias da Silva, Bharath Kumar Tirupakuzhi Vijayaraghavan

**Affiliations:** 1 Clinical Administration Apollo Hospitals Chennai India Clinical Administration, Apollo Hospitals - Chennai, India.; 2 Research Institute HCor-Hospital do Coração São Paulo SP Brazil Research Institute, HCor-Hospital do Coração - São Paulo (SP), Brazil.; 3 Hospital das Clínicas Faculdade de Medicina Universidade de São Paulo São Paulo SP Brazil Hospital das Clínicas, Faculdade de Medicina, Universidade de São Paulo - São Paulo (SP), Brazil.; 4 Department of Critical Care Medicine A. C. Camargo Cancer Center São Paulo SP Brazil Department of Critical Care Medicine, A. C. Camargo Cancer Center - São Paulo (SP), Brazil.; 5 Department of Critical Care Medicine Apollo Hospitals Chennai India Department of Critical Care Medicine, Apollo Hospitals - Chennai, India.; 6 Centre for Inflammation Research University of Edinburgh Edinburgh United Kingdom Centre for Inflammation Research, University of Edinburgh - Edinburgh, United Kingdom.

Socioeconomic status (SES) is increasingly recognized as an important determinant of both the development of critical illness and subsequent clinical outcomes.^(1)^Individuals from socioeconomically disadvantaged backgrounds often experience a higher burden of chronic disease, reduced access to preventive healthcare, and poorer overall survival.^(2,3)^Within the field of critical care medicine, growing evidence suggests that SES influences both the severity of illness at presentation and recovery after intensive care unit (ICU) admission (Figure 1). Patients with lower SES frequently present with greater physiological derangement, reflected by higher illness severity scores such as the Sequential Organ Failure Assessment (SOFA) and Simplified Acute Physiology Score (SAPS II).^(4)^ These disparities likely arise from a combination of factors, including limited health literacy, delayed care-seeking behaviour due to financial or structural barriers, and a higher baseline burden of untreated chronic comorbidities.

**Figure 1:**
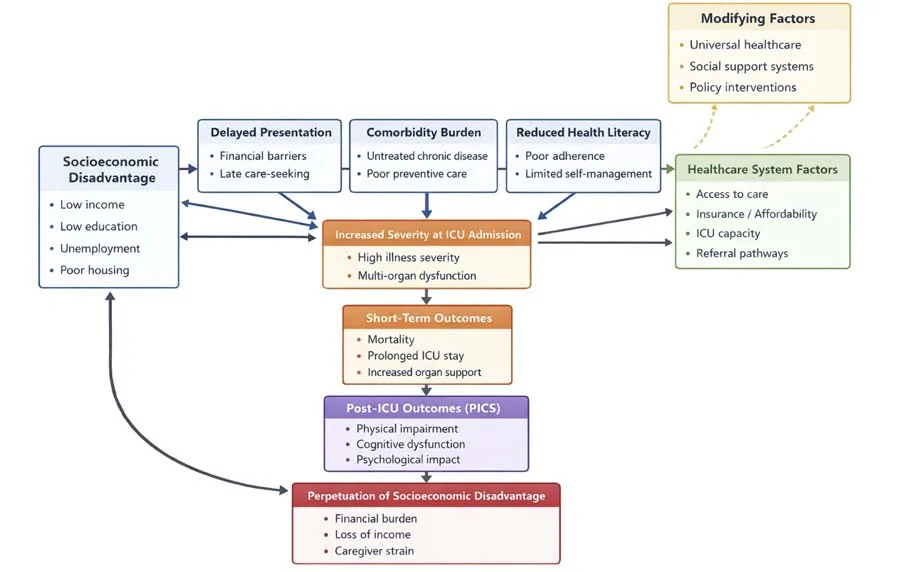
Conceptual framework illustrating the relationship between socioeconomic disadvantage and outcomes in critical illness.

ICU - intensive care unit.

Figure developed in PowerPoint by the authors, with the assistence of the artificial intelligence tool Claude.

A recent meta-analysis reported that lower SES was associated with higher short-term mortality among critically ill patients, with a 13% increase in 30-day mortality and higher SOFA score.^(1)^ These socioeconomic gradients persist even within healthcare systems that provide universal access to care. Several studies also demonstrated disparities in long-term outcomes, including higher rates of hospital readmission and an increased burden of physical, psychological, and cognitive impairments – collectively described as post-intensive care syndrome (PICS) – among survivors from disadvantaged socioeconomic groups.^(1,5,6)^

The methodology for quantifying SES varies significantly by regional economic structures and data availability, complicating global comparisons. In many countries, SES is measured using individual-level indicators such as income, education level, and employment status. In high-income countries, researchers use area-based indices derived from census data to capture neighbourhood-level resources. For instance, studies conducted in the United Kingdom utilize the Index of Multiple Deprivation (IMD), whereas research in Australia often relies on the Socio-Economic Indexes for Areas (SEIFA), including the Index of Relative Socio-Economic Advantage and Disadvantage (IRSAD). These composite indices integrate multiple indicators such as income, education, employment, and housing characteristics to estimate socioeconomic position at the population level.^(5)^ Conversely, in middle-income countries like India, composite tools, including the Modified Kuppuswamy scale and the Udai Pareek scale, are widely used. These instruments incorporate variables such as occupation and household income to assess social stratification and precipitate medical impoverishment.^(6)^ In many low-income countries where formal income data are unreliable, particularly in Sub-Saharan Africa, asset-based wealth indices are frequently employed. These indices use proxy indicators such as household assets and housing conditions to estimate socioeconomic position.^(7)^

The study by Tan et al.^(8)^ makes an important contribution to understanding the relationship between SES and critical illness. The authors should be congratulated for undertaking a large national analysis examining whether socioeconomic disadvantage influences mortality after unplanned ICU admission in Australia. Using data from the Australian and New Zealand Intensive Care Society Adult Patient Database (ANZICS APD), the investigators conducted a retrospective cohort study of over 245,000 ICU admissions between 2017 and 2019 to evaluate outcomes across socioeconomic strata defined by IRSAD. They found no association between SES and mortality after ICU admission. This study has several notable strengths. Foremost, it uses a national clinical registry encompassing a substantial proportion of ICU admissions. The investigators also used validated risk-adjustment models calibrated to the Australian ICU population and employed mixed-effects modelling to account for clustering of patients within individual ICU sites. However, several limitations warrant consideration. Socioeconomic status was measured using postcode-level indices rather than individual-level measures, obscuring heterogeneity within geographic areas and increasing susceptibility to ecological bias.

Additionally, the analysis was restricted to unplanned ICU admissions and does not capture earlier stages of the healthcare pathway, where socioeconomic disparities influence access to hospital care or referral for specialist services. Finally, the study focuses on short-term hospital mortality. It cannot evaluate long-term outcomes such as functional recovery, quality of life, or access to rehabilitation following critical illness, outcomes that may be more sensitive to SES disparities.

Taken together, these findings highlight the complex relationship between socioeconomic disadvantage and outcomes after critical illness. The absence of a significant association between SES and short-term mortality in this cohort likely reflects structural features of the Australian healthcare system, in which universal access to emergency and critical care reduces financial barriers to ICU admission and treatment. However, this may not be directly generalizable to other settings, especially low- and middle-income countries, where insurance coverage, awareness, delayed referrals, and limited critical care capacity constrain access to intensive care.

In resource-constrained healthcare settings, socioeconomic disadvantage may exert a greater influence on outcomes because of limited ICU bed availability, restricted access to advanced organ support, financial barriers to healthcare utilization, and inadequate rehabilitation and post-ICU follow-up services. As a result, disparities may arise not only in long-term recovery but also earlier in the care pathway through delayed presentation, delayed referral, and reduced access to specialized critical care services. These considerations highlight the importance of health-system interventions to improve equitable access to critical care and recovery support across diverse healthcare systems.

For the intensivist, awareness of socioeconomic disadvantage should not influence decisions regarding ICU admission, escalation of organ support, or other therapeutic interventions. Instead, recognizing socioeconomic vulnerability should prompt clinicians to identify patients at greater risk of adverse outcomes because of barriers to healthcare access, rehabilitation, medication adherence, and post-ICU follow-up. Such awareness may support targeted discharge planning, multidisciplinary support, and appropriate follow-up strategies to reduce inequities in recovery after critical illness.

Future research should examine how socioeconomic factors influence both access to critical care and post-ICU recovery across diverse healthcare systems. Studies incorporating individual-level socioeconomic indicators, evaluating earlier stages of the care pathway, and assessing other patient-centered long-term outcomes are needed, particularly in resource-constrained settings, where healthcare inequalities amplify the impact of socioeconomic disadvantage on outcomes following critical illness.
